# 

**Published:** 1823-03-01

**Authors:** 


### Contents

OF

No. 12, Vol. III. for MARCH, 1823.

ART. I.

A Treatise on Insanity, its Seat, Symptoms, Causes and Treatment. By M. GEORGET, M. D. - - - - - - - - - 701

II.

A New View of the Infection of Scarlet Fever. By WILLIAM MACMICHAEL, M. D. - - - - - - - - - - - - 724

III.

Select Dissertations on several Subjects of Medical Science. By SIR GILBERT BLANE, Bart. M.D. - - - - - - - - - 733

IV.

Practical Observations on Distortions of the Spine, Chest, and Limbs. By WILLIAM TILLEARD WARD, F.L.S. - - - 746

V.

Professors BRERA and COINDET, &c. on Iodine - - - - 757

VI.

A practical Essay on Diseases and Injuries of the Bladder. By ROBERT BINGHAM, F. R. Coll. Surg. - - - - - - 767

VII.

M. ALIBERT on Prurigo Formicans - - - - - - - - 779

VIII.

Illustrations of the Enquiry respecting Tuberculous Diseases. By JOHN BARON, M.D. - - - - - - - - - - 785

IX.

The Study of Medicine. By JOHN MASON GOOD, M.D. - - 806

X.

A Treatise on Dislocations and Fractures of the Joints. By Sir ASTLEY COOPER, Bart. - - - - - - - - - - - 832

### XI.

### PUERPERAL FEVER.

1. A Treatise on the Epidemic Puerperal Fever of Edinburgh. By W. Campbell, M.D. - - - - - - -
2. A Treatise on the Disease termed Puerperal Fever. By John Mackintosh, M. D. - - - - - - - - - - } 858

### XII.

### PHRENOLOGY.

Observations on Phrenology, as affording a Systematic View of Human Nature - - - - - - - - - - - - - - - 896

### XIII.

### QUARTERLY PERISCOPE, EXTRA.

Mr. Amesbury on the Treatment of Fractures of the Lower Extremity - - - - - - - - - - - - - - 916

### XIV.

Bibliographical Record - - - - - - - - - - - - 935

### XV.

### CORRESPONDENCE.

1. Republication of the Medico-Chirurgical Review in America 937

2. Dr. Good's Letter - - - - - - - - - - - - - - 938

3. Philo-Criticus, Answer to - - - - - - - - - - - *ib.*

4. Case of the late Mr. Knox, in Answer to the Medical Intelligencer - - - - - - - - - - - - - - *ib.*

5. Mr. Hutchinson's Letter - - - - - - - - - - - *ib.*

6. Mr. Weiss's Instruments - - - - - - - - - - - *ib.*

7. Obituary of Dr. Jenner - - - - - - - - - - 939

8. Quarterly List of Subscribers - - - - - - - - - 940

---

*In the Press,*

An EXPOSITION of the PRINCIPLES of PATHOLOGY, and of the TREATMENT of DISEASES. By Daniel Pring, M.D. Member of the Royal College of Surgeons, London.

